# Identification of RNA pseudouridine sites using deep learning approaches

**DOI:** 10.1371/journal.pone.0247511

**Published:** 2021-02-23

**Authors:** Abu Zahid Bin Aziz, Md. Al Mehedi Hasan, Jungpil Shin

**Affiliations:** 1 Department of Computer Science & Engineering, Rajshahi University of Engineering & Technology, Rajshahi, Bangladesh; 2 School of Computer Science and Engineering, University of Aizu, Aizuwakamatsu, Japan; Chuo University, JAPAN

## Abstract

Pseudouridine(Ψ) is widely popular among various RNA modifications which have been confirmed to occur in rRNA, mRNA, tRNA, and nuclear/nucleolar RNA. Hence, identifying them has vital significance in academic research, drug development and gene therapies. Several laboratory techniques for Ψ identification have been introduced over the years. Although these techniques produce satisfactory results, they are costly, time-consuming and requires skilled experience. As the lengths of RNA sequences are getting longer day by day, an efficient method for identifying pseudouridine sites using computational approaches is very important. In this paper, we proposed a multi-channel convolution neural network using binary encoding. We employed k-fold cross-validation and grid search to tune the hyperparameters. We evaluated its performance in the independent datasets and found promising results. The results proved that our method can be used to identify pseudouridine sites for associated purposes. We have also implemented an easily accessible web server at http://103.99.176.239/ipseumulticnn/.

## Introduction

Pseudouridine is the most common RNA modification observed in both prokaryotes and eukaryotes [1]. It is formed by the Ψ synthase enzyme which leads to the proof of its occurrence in various kinds of RNAs [2]. This enzyme separates the uridine residue’s base from its sugar and rotates it 180° along the N3-C6 axis. The separation is completed by the subsequent reattachment of the base’s 5’-carbon to the 1’-carbon of the sugar which results in the formation of an isomer of uridine, Pseudouridine [3]. Psudouridines play a vital role in both biological and genetic aspects of RNAs, especially for tRNA and rRNA. In case of rRNA, ribonucleoproteins are proved to be needed for pseudouridylation [4]. Psudouridines also work as a powerful mechanism for stabilizing tRNAs in both single and double-stranded regions [5]. Besides, different species present different prospects due to pseudouridines such as U6 snRNA mutants pseudouridylate at Ψ28 contributing to the filamentation growth program [6]. Furthermore, mRNAs incorporated with Ψ increase translation efficiency and restrict innate immune response [7]. Therefore, an effective method for identifying Ψ sites has a vital significance.

Some laboratory techniques have been introduced over the years producing promising results. Carlile et al. introduced a transcriptome-wide pseudouridine-seq approach where Lovejoy et al. used induced termination of reverse transcription in their work [8, 9]. Furthermore, Schwartz et al. developed a transcriptome-wide quantitative mapping system to identify pseudouridine [10]. All of these systems are not only expensive but also time consuming. Moreover, skilled and experienced people are required to maintain these systems. That is why a more user-friendly method is required for identifying pseudouridine sites.

Despite the necessity, there are not many in silico methods to identify Ψ sites from nucleotide sequences. Li et al. introduced an SVM based web server which is, as far as we know, the first computational method to identify pseudouridine synthase (PUS) specific Ψ sites [11]. They extracted features from the nucleotides around the Ψ sites which provided good results for human and yeast samples. Later, their performance was improved by taking account of the chemical properties and the occurrence frequency density distributions of nucleotides by iRNA-Pseu, proposed by Chen et al. Their work also covered another species (*M. musculus*) [12]. He et al. proposed another web server named PseUI by using SVM [13]. First, they generated five different types of features and selected one by using the sequential forward feature selection approach.

Among the recent works, Tahir et al. implemented both machine learning and deep learning methods in their work [14]. They extracted features using n-gram and MMI in their SVM classifier and adopted a convolutional neural network (CNN) in their deep learning method, where the CNN classifier produced better performance. To the best of our knowledge, this is the only method that applied deep learning methodologies for this task so far. Using the best features from forward and incremental features, Liu et al. proposed a gradient boosting based method named XG-Pseu [15]. Furthermore, Mu et al. proposed an ensemble model named iPseu-Layer consisted of three machine learning techniques [16]. They employed random forest for the final prediction.

Many of the recent works used PseKNC for feature extraction [17–19]. That is why we wanted to adopt a CNN model which does not require any additional feature extraction technique. CNN has already proven to be useful in computer vision problems. Recently CNN has been producing satisfactory results in nucleotide-based datasets [14, 20–23]. In this work, we employed a CNN model where multiple channels of convolution layers with different sized filters are applied separately. Each of these convolution layers is then added to a max-pooling layer and concatenated. Our model yielded satisfactory results in the benchmark and independent datasets.

## Materials and methods

### Dataset collection

In this work, data were collected for three different species which are *H. sapiens*, *S. cerevis*iae and *M. musculus* represented by HS, SC and MM respectively. There were three benchmark datasets, HS_990, SC_628, and MM_944, one for each species for training purposes. Each of these datasets was balanced in terms of the number of samples. These are the same datasets used in Chen et al’s work where they downloaded the RNA sequences from RMBase [12, 24]. In addition to these benchmark datasets, Chen et al. also gave two independent datasets, HS_200 and SC_200 for testing purposes which were for *H. sapiens* and *S. cerevisiae* but not for *M. musculus*. In both HS_200 and SC_200, the number of positive and negative samples was equal. In the datasets, RNA sequences were formulated as shown:

$$\begin{array}{c} R_{\xi }\left(U\right)=N_{-\xi }N_{-(\xi -1)}\cdots N_{-1}UN_{1}\cdots N_{+(\xi -1)}N_{\xi } \end{array}$$ (1)

Here, U indicates “uridine”, *N*_−*ξ*_ denotes the *ξ*-th upstream nucleotide towards the 5’ end and *N*_+*ξ*_ denotes the *ξ*-th downstream nucleotide towards the 3’ end from the central uridine. The value of *ξ* in HS_990 and MM_944 was 10 and 15 in SC_628.

### Data preprocessing

Before applying the RNA sequences to our model, we needed to preprocess it first. There was only one step involved in the preprocessing step, which was binary “one-hot” encoding to convert our inputs into a 2-dimensional matrix. Each of the nucleotides of an input sequence was represented as a row vector where all the values are zero except for one value. We applied two separate techniques for this task.

#### General “one-hot” encoding

In this technique, the length of these row vectors was four which is the number of nucleotides found in RNA. Therefore, a sequence with N nucleotides would be a (N x 4) matrix. The 1D vectors we chose for the nucleotides were: (“A” = [1, 0, 0, 0], “U” = [0, 1, 0, 0],“C” = [0, 0, 1, 0], “G” = [0, 0, 0, 1]).

#### Merged-seq “one-hot” encoding

**Fig 1:**
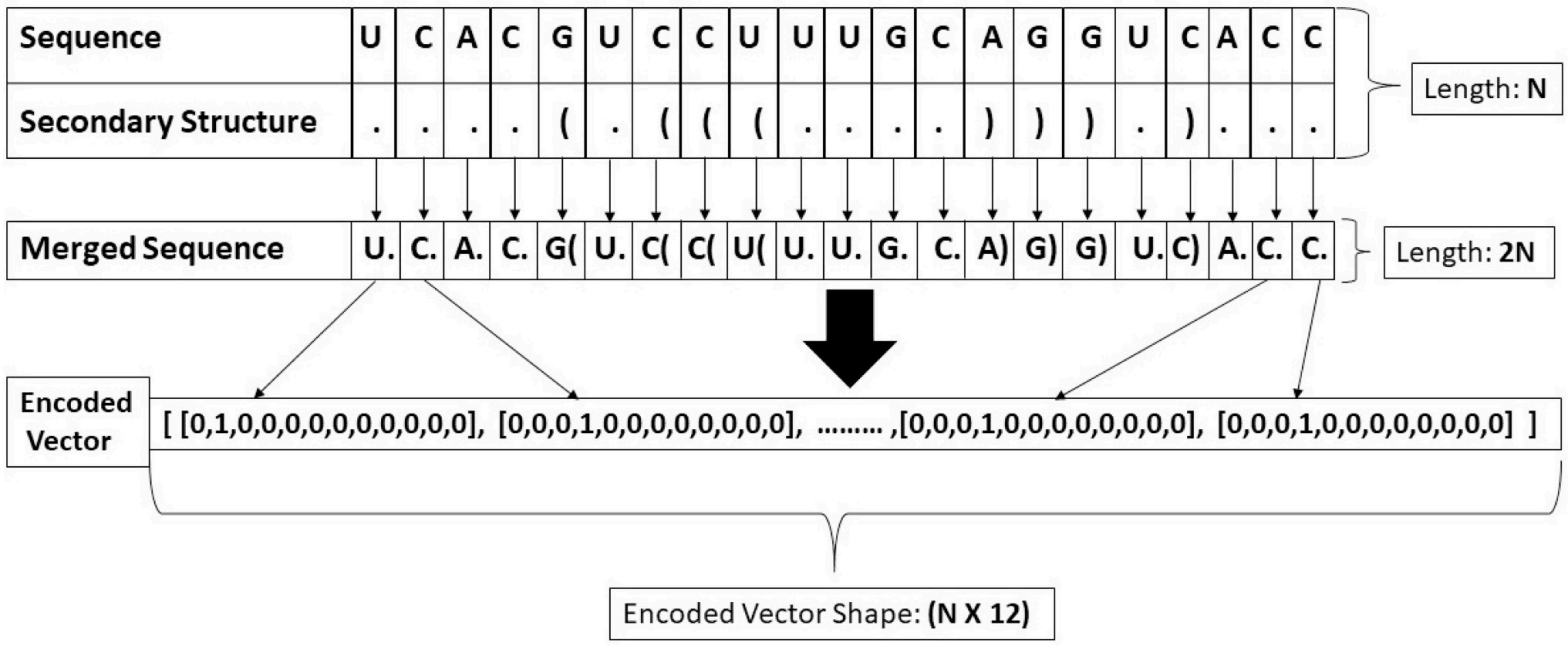
Vectorization process of the RNA sequences. Here, secondary structure was the predicted result from rnafold. Merged sequence was the pair of the original sequence and secondary structures. This merged sequence was then encoded using “one-hot” technique.

We also applied another technique by predicting secondary structures using RNAfold. Studies showed that secondary structure revealed critical structural features to detect Ψ sites [25]. We wanted to simulate this mechanism in computational methodologies. That’s why we predicted the secondary structure and merged it with the original sequence. We called it “merged-seq”. The secondary structure provided a new set of features and by merging with the original sequence we generated some more features. This technique provided good predictive performance in Zheng et al.’s pre-miRNA detection [23]. The encoding process is shown in Fig 1. The predicted secondary structure and merged sequence for each RNA sequence can be found in the supporting information or in this link: http://103.99.176.239/ipseumulticnn/datasets. The following steps were followed for this technique:

- First, we predicted the secondary structure of the original sequence using RNAfold [26]. This structure had three types of symbols: “.”, “(” and “)”. The “(” and “)” indicated that a nucleotide at 5’-end and it’s complimentary nucleotide at 3’-end is paired and the “.” indicated that the nucleotide is not paired with any other nucleotide.
- Then, we formed a merged sequence that consisted of the original sequence and the secondary structure. This merged sequence had N pairs, N being the length of the sequence. The pairs were formed by taking one nucleotide from the original sequence and one symbol from the secondary structure.
- As there were four types of bases in RNA and three types of indicators in the secondary structure, we had 12 types of pairs in the merged sequences. After that, we encoded the pairs of the merged sequences using “one-hot” technique. So after encoding, an RNA sequence of length, N became a two-dimensional matrix of (N x 12). So for both the HS and MM datasets, the preprocessed inputs turned into a (21 x 12) matrix and for the SC dataset, the inputs turned into a (31 x 12) matrix.

### CNN architecture

**Fig 2:**
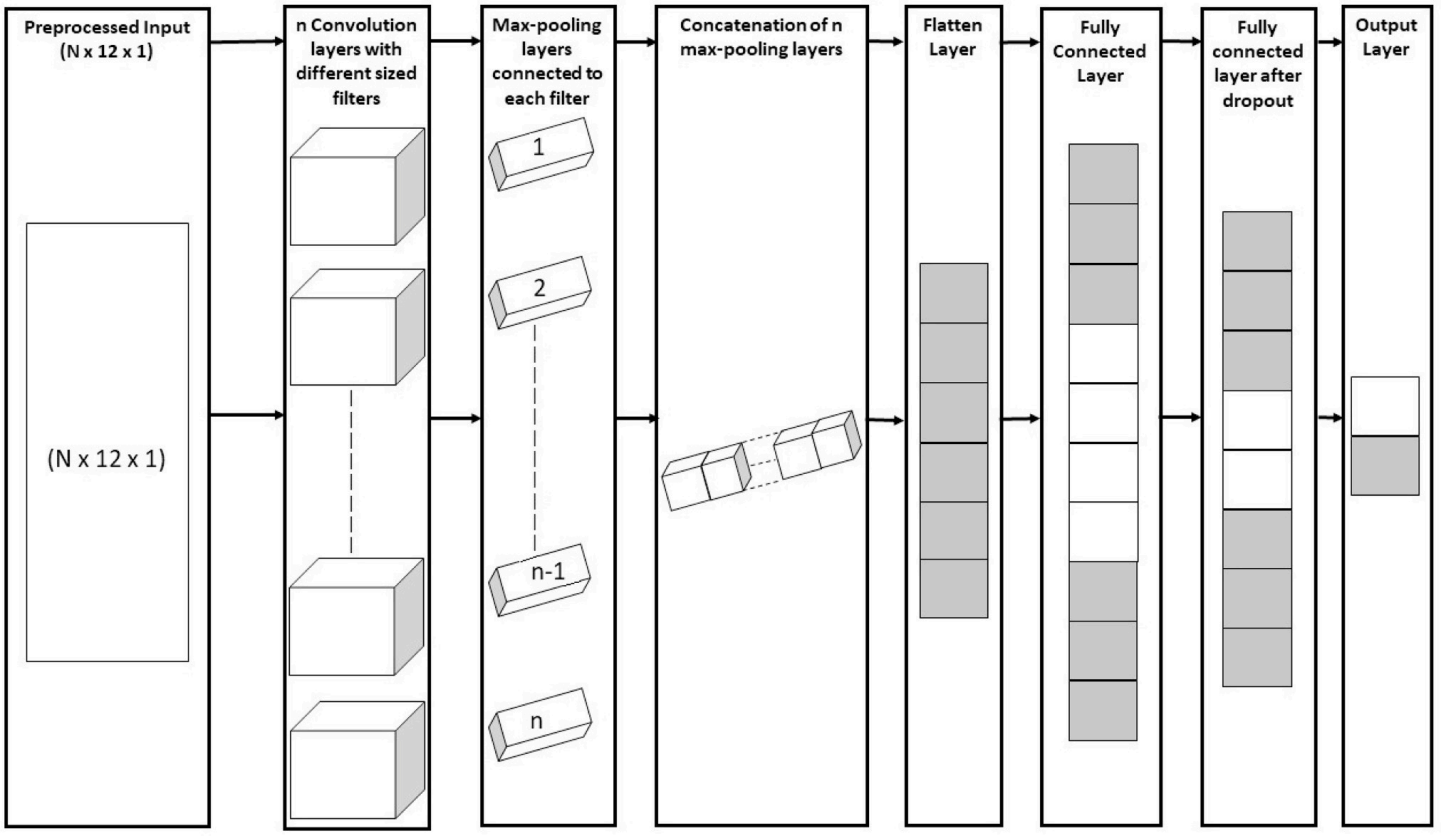
The architecture of our multi-stage CNN model.

After preprocessing (“one-hot” encoding), the converted 2D inputs were fed to a convolutional neural network. Generally, in a CNN model, the inputs are connected to some convolution and max-pooling layers, followed by a couple of fully connected layers that are connected to the output layer. But in our case, the preprocessed inputs are fed to a multi-channel CNN model which has been very effective in various text classification tasks [27, 28]. The motivation behind this approach was to make sure a sequence is processed at different lengths at a time. In a sequential model, we can use only one size of filter for each convolution which may not extract the best features all the time. That’s why we applied multiple channels of feature extraction operations(convolution and max-pooling) to the input sequence and integrated the features for better Ψ identification. A general architecture of our multi-channel model is shown in Fig 2.

Each channel of our model started with a convolution layer. We tuned the number of channels and the height of the filters of the convolution layer. The width of the filters remained unchanged. Each of these convolution layers was then connected to a max-pooling layer. Then, the max-pooling layers were concatenated together to combine the features extracted by the convolution and max-pooling layers. Next, the max-pooling layers are connected to the first fully connected layer which had 1024 nodes. After that, we employed dropout regularization to reduce the number of parameters. Then, the final layer was connected which gave a probability distribution of the classes. From the probability distribution, the final output was predicted.

The number of convolution layers was selected by applying k-fold cross-validation and grid search. Cross-validation also helped us to select the learning rate, dropout probability and height of the filters. Relu activation function was employed in every layer except for the last layer where the softmax activation function was used. This was the general structure of our model. Only the height of filters and the number of convolution layers varied for different datasets. We used categorical cross-entropy as the loss function. We also examined some well-known optimizers like Adam, Gradient descent, RMSprop etc. to minimize the loss function. Among these optimizers, Adam produced the best optimization.

### Method evaluation metrics

Four evaluation metrics have been frequently used to evaluate the quality of a method in recent studies [29–31]. To calculate them, we required four parameters: true positive (TP), true negative (TN), false positive (FT) and false negative (FN). The equations for the evaluation metrics are given below:

- Sensitivity (SN):
  $$\begin{array}{c} \text{Sensitivity}=\text{TP}/(\text{TP}+\text{FN}) \end{array}$$ (2)
- Specificity(SP):
  $$\begin{array}{c} \text{Specificity}=\text{TN}/(\text{TN}+\text{FP}) \end{array}$$ (3)
- Mathews Correlation Coefficient (MCC):
  $$\begin{array}{c} \begin{array}{c} \text{MCC}=(\text{TP}*\text{TP}-\text{TP}*\text{FN})/[(\text{TP}+\text{FP})*\\ {\left(\text{TP}+\text{FN})*(\text{TN}+\text{FP})*(\text{TN}+\text{FN})\right]}^{1/2} \end{array} \end{array}$$ (4)
- Accuracy (AC):
  $$\begin{array}{c} \text{Accuracy}=(\text{TP}+\text{TN})/(\text{TP}+\text{TN}+\text{FP}+\text{FN}) \end{array}$$ (5)

## Results and discussions

### Hyperparameter tuning

Hyperparameter tuning is vital to maximize a model’s predictive performance. On the benchmark datasets, we tuned a number of hyperparameters to fine-tune our model. We did it in three separate steps using k-fold cross-validation and grid search. We used k = 5 to compare our results with the existing works as they also applied cross-validation using the same value. This implied that we divided the benchmark datasets into 5 folds. Among them, 4 folds were used for training and the remaining fold was used for testing that particular model.

First, we tuned the number of epochs and batch size. Then, we tuned the number of channels and the height of convolution filters using the values from the first step. The number of channels was tuned to investigate how many of them can be separately connected to the input layer to produce the best accuracy. Finally, using the values from the previous steps we tuned the learning rate and dropout probability. Grid search was adopted to select the values that produced the best result.

The considered and selected values for the hyperparameters are given in Table 1. We calculated accuracies for every possible combination of values of these hyperparameters and selected the ones that provided the highest accuracy. Merged-seq “one-hot” encoding was used when we tuned the hyperparameters. Then we trained our model by applying general and merged-seq “one-hot” encoding separately using the tuned values. As the shape of the inputs were different in the datasets, the selected values were not the same. They were used to train our model in the benchmark datasets and were evaluated by the independent data.

**Table 1:** The ranges of values of the hyperparameters of the benchmark datasets.

Hyperparameters	Ranges of values	Selected values	
		SC_628	HS_990, MM_944
Batch size	[16, 32, 64, 128]	16	16
No. of epochs	[10, 50, 100, 200]	50	50
No. of channels	[5, 7, 9, 11]	7	9
Height of filters	[3, 5, 7, 9]	7	5
Learning rate	[0.0001, 0.0003, 0.0005, 0.0007, 0.001]	0.0005	0.0005
Dropout probabillity	[0.4, 0.45, 0.5, 0.55, 0.6]	0.4	0.5

### Training

Since the performance of CNN in computer vision and NLP tasks is well established, we wanted to use its classification success for biological sequence inputs. After the concatenation of the multiple convolution and max-pooling layers of our multi-stage CNN model, the number of parameters increased significantly. That is why to reduce the number of parameters, we employed dropout regularization after the first fully-connected layer. We also applied early stopping to make sure there was no overfitting in our model which means we stopped the training process if the validation loss did not improve after a certain consecutive epochs. After tuning the hyperparameters, we used the selected values to train our model in the benchmark datasets. The validation and training process were done in a core i5 laptop having NVIDIA 940m as GPU. Because of the grid search, the validation process took almost an hour to complete and the training process took about 2-3 minutes. We implemented our model using Keras Framework (2.2) with TensorFlow as backend.

We trained our model on the benchmark datasets using both general “one-hot” encoding and merged-seq “one-hot” encoding separately. Among these techniques, merged-seq “one-hot” encoding produced better performance. We employed the same model architecture in both cases using the tuned hyperparameters. We compared the performance of our models with the existing predictors (iRNA-PseU [12], PseUI [13], iPseU-CNN [14], XGboost [15], iPseU-Layer [16]) on the benchmark datasets which is shown in Table 2. From the table, we can see that our models produced satisfactory results. The training accuracy of our model was less than that of the iPseu-Layer because of their model’s overfitting which is stated by Mu et al. That’s why our model had better accuracy in the independent dataset despite having less accuracy in the benchmark datasets. Even though our model didn’t achieve the most accuracy it had increased sensitivity by 4.26% and 20.27% in SC_628 and HS_990 datasets respectively. In our case, sensitivity represents the ratio of correctly identified Ψ sites to all sequences which had Ψ sites in reality. That means our models were able to predict actual Ψ sites quite well.

**Table 2:** Comparison of the evaluation metrics with the existing predictors on the benchmark datasets.

Predictors	Benchmark Datasets											
	SC_628				HS_990				MM_944			
	AC(%)	SN(%)	SP(%)	MCC	AC(%)	SN(%)	SP(%)	MCC	AC(%)	SN(%)	SP(%)	MCC
iRNA-PseU [12]	64.49	64.65	64.33	0.29	60.40	61.01	59.80	0.21	69.07	73.31	64.83	0.38
PseUI [13]	65.13	62.74	67.52	0.30	64.24	64.85	63.64	0.28	70.44	74.58	66.31	0.41
iPseU-CNN [14]	68.15	66.84	69.45	0.37	66.68	65.0	68.78	0.34	71.81	74.79	69.11	0.44
XGboost [15]	68.15	66.84	69.45	0.37	65.44	63.64	67.24	0.31	72.03	76.48	67.57	0.45
iPseU-Layer [16]	89.34	84.68	93.76	0.79	79.70	71.18	88.22	0.60	80.08	77.92	81.82	0.60
ours(General)	81.50	76.0	87.0	0.61	77.0	79.5	74.5	0.53	80.50	86.0	75.0	0.55
ours(merged-seq)	85.85	88.29	83.37	0.72	78.83	85.61	72.07	0.59	77.23	76.62	77.60	0.54

### Comparative analysis

After training our models in the benchmark datasets, we examined its performance in the independent datasets by comparing the evaluation metrics with the existing predictors (iRNA-PseU [12], PseUI [13], iPseU-CNN [14], iPseU-Layer [16]). The findings are shown in Table 3. Similar to our training process, we tested for both general and merged-seq encoded models. Although both models produced better results than the existing predictors, the merged-seq encoded model outperformed them all.

**Table 3:** Comparison of the performance of our model with the existing predictors on the independent datasets.

Predictors	Independent Datasets							
	SC_200				HS_200			
	AC(%)	SN(%)	SP(%)	MCC	AC(%)	SN(%)	SP(%)	MCC
iRNA-PseU [12]	60.00	63.00	57.00	0.20	61.50	58.00	65.00	0.23
PseUI [13]	68.50	65.00	72.00	0.37	65.50	63.00	68.00	0.31
iPseU-CNN [14]	73.50	68.76	77.42	0.47	69.00	77.72	60.81	0.40
iPseU-Layer [16]	72.50	68.00	77.00	0.45	71.00	63.00	79.00	0.43
ours(General)	75.00	67.00	83.00	0.50	72.5	80.00	65.0	0.44
ours(merged-seq)	76.5	80.00	73.00	0.53	74.00	73.00	75.00	0.48

Among the existing methods, iPseU-CNN produced the best performance in the SC_200 dataset. So, we calculated the amount of increased performance with respect to this classifier. In the SC_200 dataset, the specificity, accuracy and MCC was increased by 6.65%, 2% and 6.38% respectively for our general “one-hot” encoded model. But for our merged-seq “one-hot” encoded model, accuracy increased by 4.08%, sensitivity increased by 16.34% and MCC increased by 12.76%. Here, our merged-seq “one-hot” encoded model produced better performance.

In the HS_200 dataset, iPseU-Layer produced the best performance among the existing methods. In this dataset, our general “one-hot” encoded model had improved performance in accuracy by 2.11%, sensitivity by 26.98% and MCC by 2.32%. On the other hand, our “merged-seq” encoded model outperformed iPseU-Layer in accuracy, sensitivity and MCC by 4.22%, 15.87% and 11.62% respectively. Similar to the SC_200 dataset, our merged-seq “one-hot” encoded model produced better evaluation metrics in this dataset.

**Fig 3:**
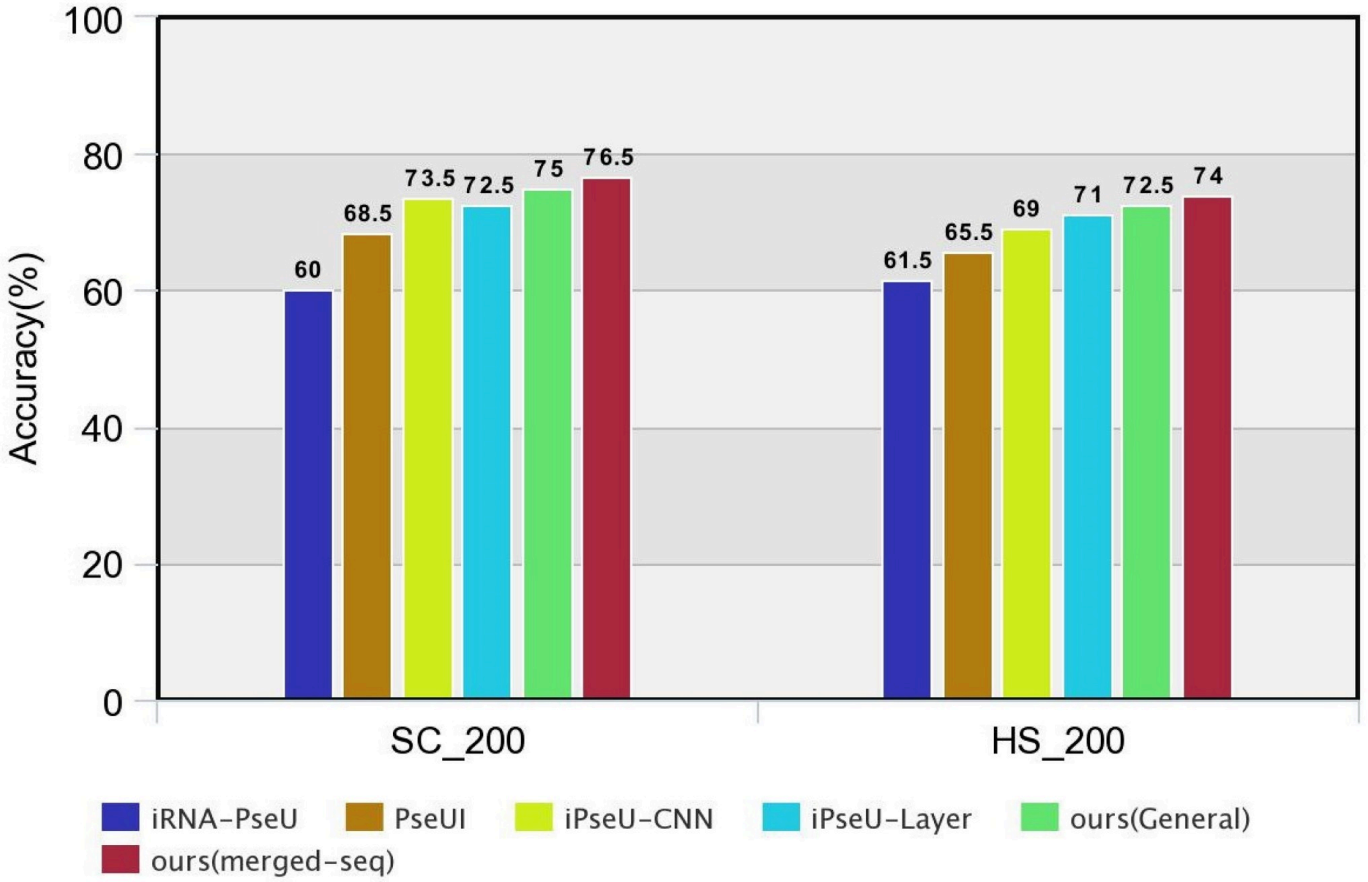
Graphical comparison of our models with the existing works in the independent datasets.

Since we applied deep learning methodologies in our work, we wanted to produce better results than other deep learning methodologies. As far as we know, iPseU-CNN is the only available deep learning methodology that used the same datasets as us. Although their encoding is similar to our general encoding technique, they adopted a single-stage sequential model where our model had multi-stage architecture. Our both general and merged-seq “one-hot” encoded model had better accuracy, sensitivity and MCC than iPseU-CNN. So we can say that our models outperform the existing deep learning methodologies in every evaluation metric. To enhance the comparison, we provided a graphical comparison of our models with the state of the art methods in the independent datasets which is depicted in Fig 3.

**Fig 4:**
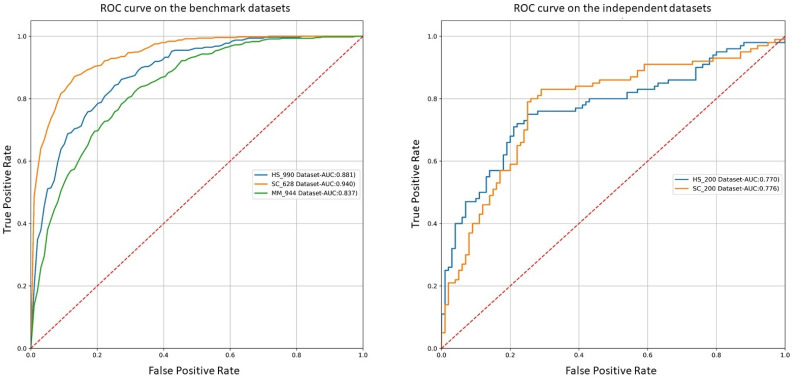
Illustration of the performance of our model in the benchmark and independent datasets using ROC curve.

We also plotted the receiver operating characteristic (ROC) curve on the benchmark and independent datasets to have a better understanding of our merged-seq “one-hot” encoded model. The plot is illustrated in Fig 4. ROC curve tells us how well a model can differentiate between classes. Our model achieved 0.88, 0.94 and 0.83 AUC (Area Under Curve) score on the HS_990, SC_628 and MM_944 benchmark datasets respectively. In case of the independent datasets, our model produced 0.77 and 0.78 on the HS_200 and SC_200 datasets respectively.

### Visualization of the learned features

**Fig 5:**
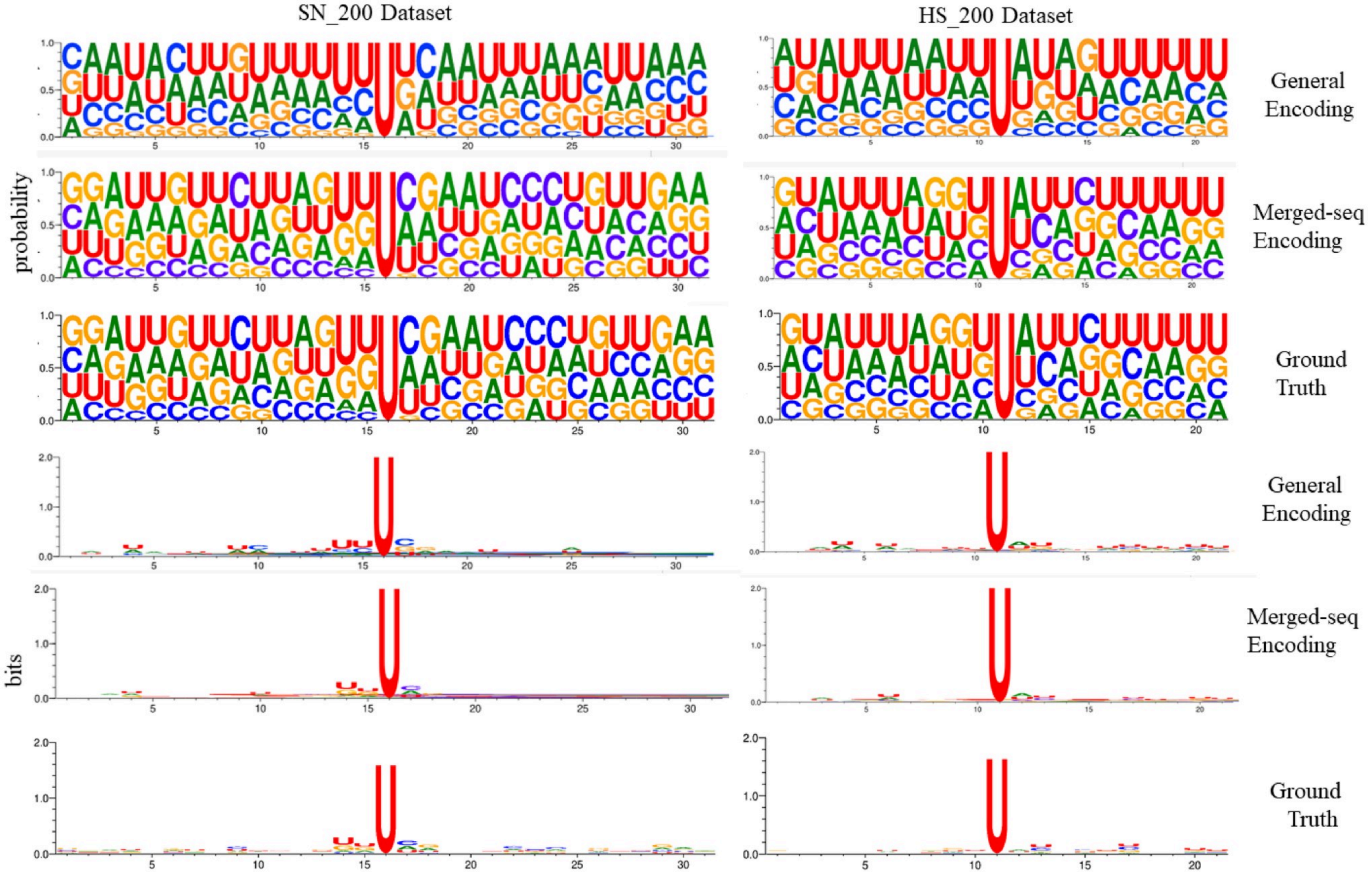
A comparison between the learned motifs of the general and merged-seq “one-hot” encoding. The ground truths were generated using Weblogo [35].

We visualized the outputs after the concatenation of the multi-stage convolution and max-pooling layers to gain further insights into the learned features for both general and merged-seq “one-hot” encoded models. We employed similar approaches used in recent CNN based works [32–34] to convert the kernel outputs into motifs. Then we used sequence logos to visualize and compare them with the logos generated from the independent datasets (Fig 5). The logos were generated in terms of probabilities (first three rows) and information contents (last three rows). From the sequence logos we can see that despite having some differences with the ground truth for the general “one-hot” encoding, the logos of the merged-seq “one-hot” encoding based models are quite similar to the ground truths. We can also observe from the information content logos that our models were able to capture the motifs around the central uracil(U) quite well for both datasets.

## Discussion

Our merged-seq “one-hot” encoded classifier is already implemented and taken to the next stage by providing a user-friendly web server. In this work, we tried to tune only those hyperparameters that can impact the performance of our classifier positively. Nevertheless, tuning other hyperparameters may result in improved performance. In our merged-seq “one-hot” encoding, the secondary structure of RNA played a vital role in improving the overall performance. We can further investigate how these new features are helping to improve the predictive performance. We also noticed some false positives for our merged-seq “one-hot” encoded model because of the secondary structure provided by RNAfold. We can investigate other secondary structure predictors in future for further improvements. We can also look for other encoding techniques of RNA sequences like Word2Vec other than “one-hot” encoding in the future. Furthermore, we can extend our work by applying our model to other species for Ψ site identification. Besides, there are other RNA modifications such as inosine (I), m3c, m5c etc. We can investigate whether our classifier can identify those sites from RNA sequences as well. Moreover, compared to the existing methods, our model produced the most accuracy in both HS_200 and SC_200 dataset.

## Conclusion

The purpose of our work was to identify pseudouridine sites from RNA sequences using computational methods, in our case, a multi-stage convolutional neural network. After preprocessing our data using “one-hot” encoding, we adopted a CNN model having multiple convolution and max-pooling layers connected to the input layer individually, which was followed by a couple of fully-connected layers and an output layer. We applied k-fold cross-validation and grid search for hyperparameter tuning. We trained our model by using the selected values from tuning. Then we tested the performance of our model using the independent datasets and found 74% accuracy in the HS_200 dataset and 76.5% accuracy in the SC_200 dataset. It is projected that our classifier can become a helpful tool for identifying Ψ sites. We can also say that CNN can be used as an important method for classifying biological data.

## Supporting information

S1 File The benchmark and independent datasets with the secondary structure by RNAfold and merged sequence that we applied in this work. (ZIP)

S2 File Probabilities of each sequence of the independent datasets. (ZIP)
